# Impact of sex, ancestry, and local ancestry on Apolipoprotein E (APOE) for Alzheimer’s disease (AD): a pooled case‐control analysis of Alzheimer’s Disease Genetics Consortium (ADGC)

**DOI:** 10.1002/alz.087081

**Published:** 2025-01-03

**Authors:** Omar Garcia Rodriguez, Lily Wang, Juan I Young, Alzheimer’s Disease Genetics Consortium (ADGC), Margaret A Pericak‐Vance, Eden R. Martin, Brian W. Kunkle

**Affiliations:** ^1^ University of Miami Miller School of Medicine, Miami, FL USA; ^2^ Department of Public Health Sciences, University of Miami Miller School of Medicine, Miami, FL USA; ^3^ Dr. John T. Macdonald Foundation Department of Human Genetics, University of Miami Miller School of Medicine, Miami, FL USA; ^4^ John P. Hussman Institute for Human Genomics, Miller School of Medicine, Miami, FL USA; ^5^ University of Pennsylvania, Philadelphia, PA USA; ^6^ Perelman School of Medicine, University of Pennsylvania, Philadelphia, PA USA; ^7^ 1501 NW 10th Avenue, Miami, FL USA; ^8^ John P. Hussman Institute for Human Genomics, Miller School of Medicine, University of Miami, Miami, FL USA

## Abstract

**Background:**

Previous studies have reported that non‐Hispanic white (NHW) females carrying the APOE ε4 allele differ in risk of developing Alzheimer’s disease (AD) when compared to men. Recent studies suggest the association between APOE ε4 and AD risk may be modified by age and its local ancestry in admixed populations. However, there is still scant evidence on how sex could interact with these factors. We aimed to investigate the association of sex, ancestry, APOE, and local ancestry with AD in multiple cohorts.

**Methods:**

A pooled case‐control study was undertaken from 50 independent datasets (n = 30,539;age≥55years) in the Alzheimer’s Disease Genetics Consortium. Logistic regression models were used to study the associations between disease status and independent variables including age, sex, reported race (as a proxy of ancestry), and APOE genotype. Additionally, mixed models were implemented for the local ancestry APOE haplotype analyses among different ethnicities. A p‐value less than 0.05 was considered statistically significant.

**Results:**

Regardless of ancestry, men and women with APOE ε3/ε4 or ε4/ε4 genotypes showed no significant difference in the risk of developing AD. When comparing subjects with APOE ε3/ε4 or ε4/ε4 (vs. ε3/ε3), East‐Asian (EA) males (*OR*,5.56; 95% CI:4.26‐7.60) and females(*OR*,5.46:4.34‐6.86) had the strongest ORs; whilst the weakest risk was for South‐Asian (SA) men(*OR*,1.14:0.99‐2.57) among all races/ethnicities. Age‐stratified analyses showed that NHW females(*OR*,5.36:4.74‐6.07 vs. males: OR,4.72:4.10‐5.60) had a higher risk of developing AD between ages 66‐75; whereas African American (AA), EA and SA exhibited this pattern at 55‐64. In Hispanics, the relationship is reversed, with males aged 55‐65 years carrying APOE ε3/ε4 or ε4/ε4(*OR*,4.53:1.67‐12.30: vs. females: *OR*,3.03:1.59‐5.78) having a stronger association with AD risk than their counterpart. No differences were found when stratified by sex and age for APOE ε2 and AD. Local ancestry (European ‐EU‐; African ‐AF‐; Amerindian ‐AI‐; and EA) analyses are being conducted by ancestry and sex.

Preliminary analyses suggest differences by sex and local ancestry for APOE ε4.

**Conclusions:**

Our findings suggest differences by sex and reported ancestry in the risk for developing AD in carriers of APOE ε4. Global, local ancestry and sex should be considered in predicting risk due to APOE alleles.

